# Insurance disparity in cardiovascular mortality among non-elderly cancer survivors

**DOI:** 10.1186/s40959-021-00098-8

**Published:** 2021-03-20

**Authors:** Tiantian Shi, Changchuan Jiang, Cenjing Zhu, Fangcheng Wu, Irma Fotjhadi, Stuart Zarich

**Affiliations:** 1 Department of Medicine, Bridgeport Hospital, Bridgeport, CT USA; 2 Department of Medicine, Roswell Park Comprehensive Cancer Center, Buffalo, NY USA; 3 Department of Chronic Disease Epidemiology, School of Public Health, Yale University, New Haven, CT USA; 4 Department of Medicine, Memorial Hospital West, Pembroke Pines, FL USA; 5 Division of Cardiovascular Medicine, Bridgeport Hospital, 267 Grant St, Bridgeport, CT 00610 USA

**Keywords:** Insurance disparities, Cancer survivorship, Outcomes, Cardio-oncology

## Abstract

**Background:**

Insurance status plays a vital role in cancer diagnosis, treatments and survival. Cancer patients have higher cardiovascular disease (CVD) mortality than the general population.

**Methods:**

The Surveillance, Epidemiology and End Results (SEER) program 2007–2016 was used to estimate the CVD mortality among cancer patients aged 18 to 64 years at the time of diagnosis of an initial malignancy with the eight most prevalent cancers. Standardized mortality ratios (SMRs) were calculated for each insurance (Non-Medicaid vs Medicaid vs Uninsured) using coded cause of death from CVD with adjustment of age, race, and gender. The Fine-Grey Model was used to estimate adjusted Hazard Ratios (HR) of each insurance in CVD mortality.

**Results:**

A total of 768,055 patients were included in the final analysis. CVD death in patients with Medicaid insurance remained higher than in those with Non-Medicaid insurance (HR = 1.71; 95%CI, 1.61–1.81; *p* < 0.001). Older age, male gender, and black race were all associated with increased CVD mortality in the multivariable model. Compared to the general population, patients with Medicaid had the highest SMRs of CVD mortality, regardless of year of cancer diagnosis, follow-up time, cancer site, and race. Non-Medicaid insured patients had similar CVD mortality to the general population after 2 years out from their cancer diagnosis.

**Conclusion:**

Cancer patients with Non-Medicaid insurance have significantly lower CVD mortality than those with no insurance or Medicaid. The insurance disparity remained significant regardless of type of CVD, cancer site, year of diagnosis and follow-up time.

**Supplementary Information:**

The online version contains supplementary material available at 10.1186/s40959-021-00098-8.

## Introduction

Cardiovascular diseases (CVD) is the leading cause of death in United States, followed closely by cancer. In 2017, 23% of all deaths were due to CVD, while 21.3% of deaths were due to malignant neoplasms [1]. Due primarily to advancements in cancer screening and treatment, the cancer survivor population has been growing rapidly in the U.S. from 15.5 million cancer survivors in 2016 to a projected 26.1 million by 2040 [2]. There is an urgent call to understand the needs and challenge faced by this emerging patient population, especially as it relates to novel cancer therapies and their cardiovascular side effects.

Currently more cancer survivors are dying from non-cancer etiologies including CVD. Cancer and CVD share many risk factors including smoking, alcohol intake, and mental distress [3]. Additionally, chemotherapy, radiation and some immune targeting therapies may increase the CVD risk during or after cancer treatment [4]. Recent studies have shown that CVD mortality risk in cancer patients is significantly higher than that of their healthy peers, especially within the first year after cancer diagnosis [5]. This CVD mortality risk remains significantly higher 7 years after breast cancer diagnosis as compared to the general population, which suggests cancer and its treatment may have long-term consequences on cardiovascular system [6].

It is well known that insurance status plays a vital role in cancer survival. Patients without Non-Medicaid insurance were less likely to receive guideline-concordant treatment, early detection and or screening [7], and were more likely to have adverse outcomes. Medicaid or uninsured status (MUS) was independently associated with a higher risk of death from cancer and delayed cancer diagnosis [8]. MUS patients at the time of diagnosis were 1.6 times as likely to die in 5 years as compared to those who had Non-Medicaid insurance [7]. Less is known regarding insurance status and its effects on CVD mortality, however it is a reasonable assumption that cancer patients with relatively poor insurance may receive suboptimal cardiovascular care or surveillance.

Direct evidence is still limited regarding the role of insurance status on CVD mortality among cancer patients. In a preliminary study, patients with Non-Medicaid insurance had better CVD outcomes in early stage lung cancer, with Medicaid patients experiencing twice the CVD mortality of Non-Medicaid insured patients [9]. Our study explores the association of insurance status with cardiovascular outcomes in cancer survivors using the population-based Surveillance, Epidemiology, and End Results (SEER) program registry [10].

## Methods

The study was exempt from Institutional Review Board review because the dataset is publicly available and de-identified. The SEER program was utilized to examine the effects of insurance status on CVD mortality in a non-elderly cohort in our study. SEER is a registry representing 28% of the US population based on yearly tumor census data, including patient demographics, primary tumor site, stage at diagnosis, initial course of treatment, follow-up time, and survival [11]. The SEER registry has also collected data on patient’s insurance status since 2007, but is not validated for patients aged more than 65 years old due to Medicare eligibility.

Participants were included (supplementary figure) if they met all of the following criteria 1) aged 18 to 64 years at the time of diagnosis of an initial malignancy; 2) having the following cancers (colorectal, lung, breast, ovarian, cervical, bladder cancer, Hodgkin’s lymphoma and Non-Hodgkin’s lymphoma); 3) diagnosis of cancer after 2007. The exclusion criteria included 1) lack of insurance information, 2) lack of information on cause of death.

Insurance status was defined as insured or insured without specifics, Medicaid Insurance (any Medicaid, including Indian Health Service), or uninsured. The definition for insured in SEER included Non-Medicaid insurance, Medicare, or military coverage at the time of diagnosis and will be referred as Non-Medicaid insurance going forward for simplification purposes. Race was classified as Hispanic and non-Hispanic White, non-Hispanic Black, Asian and Others. We categorized patients into three groups by the year of diagnosis (2007–2010, 2011–2013, 2014–2016) to assess changes in insurance status prior to and after implementing the Affordable Care Act (ACA). The extent of disease was categorized as in situ, local (no nodal or metastatic disease), region (nodal disease), or distant (any metastatic disease). The initial course of treatment was categorized based on whether patients received chemotherapy, radiotherapy or surgery as binary variables for each treatment.

Cause of death was defined by the International Classification of Diseases (ICD-9) code based on death certificates. CVDs was defined as heart disease, hypertension, cerebrovascular disease, atherosclerosis, aortic aneurysm/dissection, and other diseases of arteries, arterioles, or capillaries.

Patient and clinical characteristics were compared using the Chi-Square test. A competing risk model was conducted and communicative incidence function was calculated with the Fine and Grey model [12]. We also used the Fine-Grey model to compare the risk of death from CVD among different insurance groups, after adjusting other risk factors including age, gender, marital status, race/ethnicity, year of diagnosis, if patient received radiation therapy, chemotherapy or surgery respectively, and cancer sites. The proportional hazards assumption was confirmed by inspection of log (−log [CVD death] curves).

To further illustrate the risk of death from CVD in cancer patients in different insurance group, we used standardized mortality ratios (SMRs) to provide the relative risk of death from CVD for cancer survivors as compared to the standard US population, adjusted by age, race, and sex over the same time. We describe the risk of death from CVD as a function of age at cancer diagnosis, year of cancer diagnosis, and follow up time, respectively. We also describe the risk of CVD mortalities by cancer site. The reference cohort was US mortality as reported in the National Vital Statistics System and maintained by the National Center for Health statistics. The SMRs were calculated using SEER*Stat 8.3.6. and all other statistical analyses were performed using SAS 9.4 software and Microsoft Excel 16.0.

## Results

### Patient characteristics

A total of 768,055 patients were included in the study. Patient demographics and clinical characteristics are shown in Table 1 and Table 2. The mean age of our cohort was 52.9 + 9.0 years and consisted of 66% white, 13% black, and 21% Asian/other races. The majority of patients were female (72.4%) and single (61.4%). Blacks and other race patients were less likely to have Non-Medicaid insurance as compared to white patients (68.4% vs 83.8% *p* < 0.0001). Medicaid and uninsured status (MUS) patients were younger, more likely to be married, and less likely to receive surgical intervention for their cancer (all *p* < 0.001) as compared to patients with Non-Medicaid insurance. MUS was associated with a striking increase in patients presenting with metastatic disease (35.7% vs 23.1%; *p* < 0.0001), as well as a much higher incidence of lung cancer (26.7% vs 17.4%; *p* < 0.0001) as compared to patients with Non-Medicaid insurance. On the contrary, patients with Non-Medicaid insurance had a higher incidence of breast cancer as compared to MUS patients (47.4% vs 35.1%; p < 0.0001). Finally, as compared to the pre-ACA era (2007–2010), by 2014–2016 there was a significant decrease in uninsured status (3.3% vs 5.2%) and an increase in Medicaid insured status (14.7% to 19.25), both *p* < 0.0001.

**Table 1:** Socio-demographic characteristics of Cancer Patients by Health Insurance Coverage, from SEER 2007–2016

	Total	Non-Medicaid	Medicaid	Uninsured	***p***-Value^*^
**N%**	768,055 (100%)	602,940 (78.5%)	128,901 (16.8%)	36,214 (4.7%)	
**Gender**					< 0.001
Male	212,206 (27.6%)	160,972 (26.7%)	36,865 (28.6%)	14,369 (39.7%)	
Female	555,849 (72.4%)	441,968 (73.3%)	92,036 (71.4%)	21,845 (60.3%)	
**Age (Mean +/− SD)**	52.9 ± 9.0	53.2 ± 8.8	52.0 ± 9.4	51.9 ± 9.5	< 0.001
**Race**					< 0.001
White	505,097 (65.8%)	423,016 (70.1%)	62,672 (48.6%)	19,409 (53.6%)	
African American	98,942 (12.9%)	65,997 (19.6%)	25,306 (21.1%)	7639 (7.7%)	
Others	164,016 (21.3%)	113,927 (18.9%)	40,923 (31.8%)	9166 (25.3%)	
**Marital Status**					< 0.001
Married	296,431 (38.6%)	192,854 (32.0%)	82,636 (64.1%)	20,953 (57.9%)	
Single	471,624 (61.4%)	410,088 (68.0%)	46,275 (35.9%)	15,261 (42.1%)	

^*^ *p*-value were estimated using chi-sq tests for all variables except for age. *P*-value for age was estimated using ANOVA test

**Table 2:** Clinical characteristics of Cancer Patients by Health Insurance Coverage, from SEER 2007–2016

	Total	Non-Medicaid Insurance	Medicaid	Uninsured	***p***-Value^*^
**Year of Diagnosis**					< 0.001
2007–2010	302,705	242,494 (80.1%)	44,527 (14.7%)	15,684 (5.2%)	
2011–2013	230,397	178,237 (77.3%)	39,321 (17.1%)	12,839 (5.6%)	
2014–2016	234,953	182,209 (77.5%)	45,053 (19.2%)	7691 (3.3%)	
**Therapy**					< 0.001
Radiation therapy	294,652	233,250 (38.7%)	49,626 (38.5%)	11,776 (32.5%)	
Chemotherapy	412,782	321,439 (53.3%)	71,491 (55.5%)	19,852 (54.8%)	
Surgery	544,696	448,781 (74.4%)	77,056 (59.8%)	18,859 (52.1%)	
**Extent of cancer**					< 0.001
in situ	23,904 (3.1%)	20,640 (3.4%)	2302 (1.8%)	962 (2.7%)	
Localized	32,536 (42.4%)	272,769 (45.2%)	42,813 (33.2%)	9784 (27.0%)	
Regional	220,740 (28.7%)	170,364 (28.3%)	40,222 (31.2%)	10,154 (28.0%)	
Distant	198,045 (25.8%)	139,167 (23.1%)	43,564 (33.8)	15,314 (42.3%)	
**Site of cancer**					< 0.001
Colorectal	9369 (12.2%)	72,171 (12.0%)	15,352 (11.9%)	6167 (17.0%)	
Lung	149,075 (19.4%)	105,051 (17.4%)	33,410 (25.9%)	10,614 (29.3%)	
Breast	343,516 (44.7%)	285,563 (47.4%)	49,231 `1 8 .2%)	8722 (24.1%)	
Cervical	25,714 (3.4%)	15,329 (2.5%)	8344 (6.5%)	2041 (5.6%)	
Ovarian	29,383 (3.8%)	22,891 (3.8%)	4688 (3.6%)	1804 (5.0%)	
Urinary/bladder	43,890 (5.7%)	82.60 (6.0%)	5455 (4.2%)	2180 (6.0%)	
Hodgkin lymphoma	1498 (2.0%)	11,488 (1.9%)	2458 (1.9%)	1034 (2.9%)	
Non-Hodgkin lymphoma	67,807 (8.8%)	54,192 (9.0%)	9963 (7.7%)	3652 (10.1%)	

^*^ *p*-value were estimated using chi-sq tests

### Outcomes

#### Cardiovascular mortality

In our cohort, 1.1% patients died as a result of CVD. Among CVD deaths the majority were due to heart diseases (79.5%) and cerebrovascular diseases (14.0%) with hypertension without heart disease, aortic aneurysm and dissections, and atherosclerosis accounting for the majority of remaining causes for CVD deaths. The frequencies of cardiovascular death varied significantly according to insurance status (*p* < 0.001), with the highest proportion of deaths occurring in Medicaid insured (1.65%) and proportionally fewer deaths among uninsured (1.28%) and Non-Medicaid insured patients (0.91%).

#### Multivariable proportional hazard model

After adjusting for demographic and tumor factors, the hazard of CVD death in patients with Medicaid insured patients remained much higher than that in patients with Non-Medicaid insurance (HR = 1.71; 95%CI, 1.61–1.81; *p* < 0.001) (Table 3). Patients without insurance showed a similar trend in death from CVD compared to patients with Non-Medicaid insurance, but this was not statistically significant (HR = 1.08; 95%CI, 0.98–1.19; *p* = 0.11) perhaps due to the small number of patients in this group. Similar multivariable models showed Medicaid and Uninsured patients had significantly higher risks in cancer-specific and all-cause mortalities (all *p* < 0.001) as compared to patients with Non-Medicaid insurance.

**Table 3:** Multivariable Fine-Grey model for CVD mortality, Cancer-specific mortality and All-cause mortality

Insurance status	HR	95% CI		P
**CVD mortality**				
Non-Medicaid insured	–	–	–	
Medicaid insured	1.71	1.61	1.81	< 0.001^a^
Uninsured	1.08	0.98	1.19	0.111
**Cancer-specific mortality**				
Non-Medicaid insured	–	–	–	
Medicaid insured	1.24	1.23	1.26	< 0.001^a^
Uninsured	1.25	1.22	1.27	< 0.001^a^
**All-cause mortality**				
Non-Medicaid insured	–	–	–	
Medicaid insured	1.44	1.42	1.45	< 0.001^a^
Uninsured	1.37	1.35	1.39	< 0.001^a^

*HR* hazard ratio

*CI* confidential interval

^a^: Variables were statistically significant in the multivariable fine-grey models, adjusted with patient’s current age, gender, racial group, marital status, year of diagnosis, age of diagnosis, extent of cancer, cancer site, treatments including radiation therapy, chemotherapy, and surgery

Table 4 further explores CVD mortality by insurance status and other major clinical variables. In multivariable analysis older age, male gender, and black race were all associated with an increased hazard of cardiovascular death. An increased hazard of CVD mortality was also seen in patients of certain tumor sites (lung, urinary and bladder), while breast cancer conferred a lower hazard for CVD mortality. Interestingly, other race patients had a significantly lower hazard for CVD mortality which is in keeping from CDC data showing that Hispanic and Asian patients have a lower age-adjusted death rate as compared to both black and white race patients [1].

**Table 4:** Multivariable Fine-Grey model for cardiovascular mortality

Parameter	HR	95% CI		p
**Insurance status**				
Non-Medicaid insured	–	–	–	–
Medicaid insured	1.71	1.61	1.81	< 0.001^a^
Uninsured	1.08	0.98	1.19	0.112
**Year of diagnosis**				
2007–2010	–	–	–	–
2011–2013	0.787	0.748	0.83	< 0.001^a^
2014–2016	0.624	0.584	0.67	< 0.001^a^
**Age of diagnosis**				
20–39	–	–	–	–
40–54	2.92	2.45	3.47	< 0.001^a^
55–64	6.13	5.16	7.28	< 0.001^a^
**Race**				
White	–	–	–	–
Black	1.51	1.43	1.60	< 0.001^a^
Other races	0.73	0.68	0.79	< 0.001^a^
**Cancer site**				
Colorectal	–	–	–	–
Lung	1.23	1.13	1.33	< 0.001^a^
Breast	0.67	0.62	0.73	< 0.001^a^
Cervical	1.12	0.95	1.31	0.174
Ovarian	0.83	0.70	0.97	0.018^a^
Urinary/bladder	1.37	1.22	1.53	< 0.001^a^
Hodgkin lymphoma	1.20	0.98	1.47	0.071
Non-Hodgkin lymphoma	0.99	0.89	1.09	0.819

Models were adjusted for patient’s gender, marital status, extent of cancer, cancer site, treatments including radiation therapy, chemotherapy, and surgery

^a^: Variables were tested statistically significant in multivariable Fine-Grey models

#### Standardized mortality ratios (SMR)

SMRs for the six types of cardiovascular disease among cancer patients were listed in Table 5 by insurance type. Medicaid insured cancer patients had the highest CVD SMR (3.57) among the three insurance groups, while patients with Non-Medicaid insurance had the lowest SMRs (except for atherosclerosis as compared to uninsured patients). Regardless of the specific subtypes of CVD, Medicaid insured patients had a statistically higher mortality as compared to the general population (Table 5).

**Table 5:** Standard Mortality Ratio (SMR) of Cardiovascular Diseases by Type of CVD in Cancer Patients with Different Insurance Status

	Non-Medicaid insured	Uninsured	Medicaid
All CVD	1.26^a^	2.23^a^	3.57^a^
Diseases of Heart	1.28^a^	2.37^a^	3.69^a^
Hypertension without Heart Disease	1.13	1.21	3.62^a^
Cerebrovascular Diseases	1.22^a^	1.77^a^	2.91^a^
Atherosclerosis	1.52	1.37	7.50^a^
Aortic Aneurysm and Dissection	0.90	2.43	2.79^a^
Other Diseases of Arteries, Arterioles, Capillaries	1.43^a^	2.53	3.56^a^

^a^ Statistically higher than the general US population

**Fig. 1:**
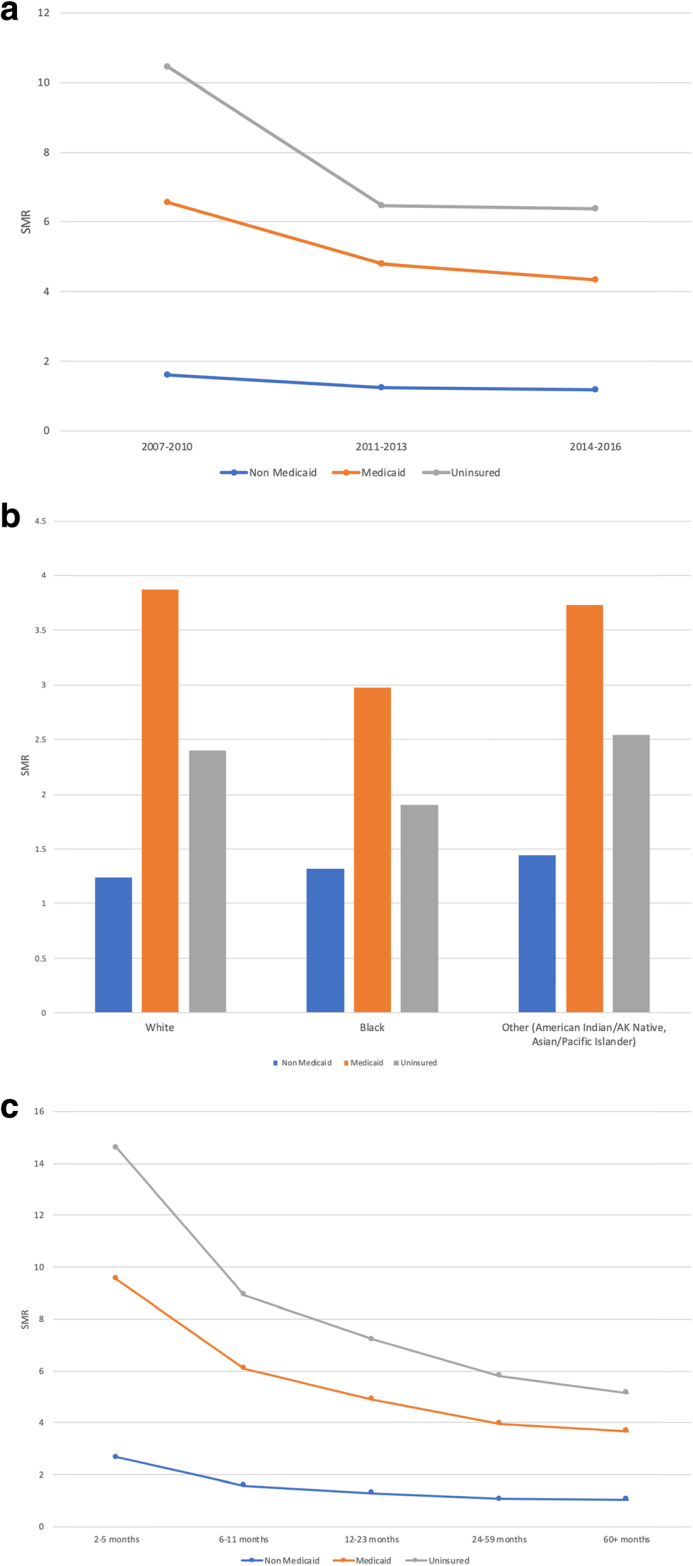
**a** Standard Mortality Ratio (SMR) of Cardiovascular Diseases by Year of Cancer Diagnosis in Patients with Different Insurance Status. **b** Standard Mortality Ratio (SMR) of Cardiovascular Diseases by Race in Patients with Different Insurance Status. **c** Standard Mortality Ratio (SMR) of Cardiovascular Diseases by Follow Up Time Diagnosis in Patients with Different Insurance Status. SMR was estimated using SEER*Stat 8.3.6. SMR of all groups were statistically higher than the general population (the reference group)

When stratified by year of cancer diagnosis (Fig. 1a) and follow-up time (Fig. 1c), patients with Medicaid insurance consistently had the highest SMR compared to the other two groups. The CVD mortality rate among the cancer patients was significantly higher than that of the standardized population for each insurance strata. Over time CVD risk decreased from 2001 to 2016 for all insurance groups as compared to the general population which may be due to improved cancer therapies and the advent of cardio-oncology awareness. Although implementation of the ACA improved insurance coverage, the disparity between both Medicaid and uninsured status as compared to Non-Medicaid insurance still persists.

Similar outcomes were noted when cardiovascular SMR rates were stratified by race (Fig. 1b). Medicaid beneficiaries had the highest SMR among other groups regardless of their racial and ethnic identities. White Medicaid beneficiaries had the highest SMR of 3.87 and Non-Medicaid insured white patients had the lowest SMR of 1.24. Black Medicaid beneficiaries comparatively had the lowest SMR as compared to the other ethnic groups.

CVD mortality decreases across all insurance strata as patients transitioned from active cancer treatment to longer term survivorship (Fig. 1c). Again, CVD mortality through survivorship was highest in the Medicaid population. After 24 months patients with Non-Medicaid insurance had similar CVD as compared to the general population. In contrast after 5 years the risk of CVD death was still 2.6 times higher in the Medicaid group as compared to the general population.

**Fig. 2:**
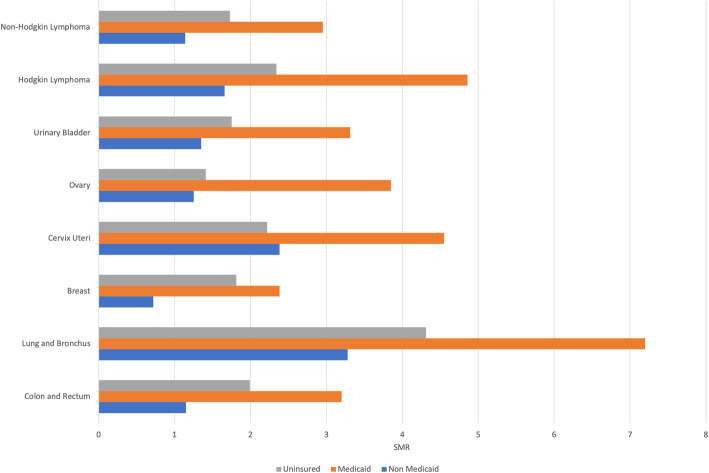
Standard Mortality Ratio (SMR) of Cardiovascular Diseases by Cancer Site in Patients with Different Insurance Status

When stratified by site of cancer (Fig. 2), patients in each subgroup had a significantly higher risk of CVD mortality compared to the general population. Lung and bronchus cancer patients who are Medicaid beneficiaries had the highest SMR (7.20), and breast cancer patients with non-Medicaid insurance had the lowest SMR (0.72) compared to other groups. Again, the marked disparity between Medicaid and Non-Medicaid insurance persists across cancer subtypes.

Interestingly, breast cancer patients had a lower risk of CVD mortality, even after adjustment for gender, despite likely higher exposure to more cardiotoxic therapies (anthracyclines and/or chest compared to the general populations (except for Non-Medicaid group), which is consistent with previous study [5]. However, the majority of breast cancer patients (64.4% according to Seer data) were diagnosed at an early localized stage and may only need hormonal therapy after surgery, which is not particularly cardiotoxic. Additionally, breast cancer patients are less likely to have CVD risk factors such as smoking exposure as compared to patients with cervical, bladder, or lung malignancies.

## Discussion

In this population-level study of cancer survivors, we analyzed the association of insurance status with CVD mortality among non-elderly adult patients with any one of the 8 most common cancers. We found that cancer patients with Non-Medicaid insurance have significantly lower CVD mortality than those with MUS. In addition, we found that the insurance disparity remained significant regardless of type of CVD, cancer site, year of diagnosis or follow-up time. To our best knowledge, this is the first study to highlight insurance disparity in CVD mortality among non-elderly adult cancer survivors and it also points out the role of insurance in multidisciplinary care throughout survivorship, as cancer patients remain at higher risk of CVD mortality.

Our results were consistent with previous study results from the general population: patients with no or Medicaid insurance are at higher risks for CVD mortality. We found the SMR of CVD in the Non-Medicaid insurance group was significantly lower than uninsured and Medicaid insured groups, regardless of type of CVD. Similarly, in the general population, lack of health insurance was independently associated with in-hospital mortality in STEMI hospitalizations (OR 1.77, 95% CI 1.72 to 1.82) according to a retrospective cohort study [13]. Similarly, advanced heart failure patients with Medicaid are also less likely to receive left ventricular support devices and Medicaid is a significant predictor of higher one-year mortality in this group [14]. Our study also echoes well-known insurance disparities in cancer treatment. No insurance or Medicaid insurance is associated with delayed diagnosis, a lower likelihood of receiving standard of care therapy, and worse cancer-specific survival [15]. Despite the difference in countries, our results are similar to that of childhood cancer survivors in Finland where patients with public insurance, as well as uninsured patients, had higher all-cause mortality (HR = 1.54) and CVD mortality (HR = 1.62)) as compared to Non-Medicaid insured patients after adjusting for demographic and lifestyle risk factors [16].

In addition, our study found that insurance status, even with adjustment for age, race, gender, marital status, cancer stage, cancer site, and treatment is an independent risk factor for CVD mortality among cancer patients. This finding may well be explained by the lower socioeconomic status, prevalent financial hardship, lower health literacy, lower compliance to medications due to various reasons, and limited access to high-quality survivorship care among patients with no or Medicaid insurance [17, 18]. This may help to explain the large difference in the number of patients presenting late in their course with metastatic disease in patients with MUS. Cancer also shares many risk factors with CVD including smoking, excessive alcohol intake, and a sedentary lifestyle. Cancer patients who lack Non-Medicaid insurance have remarkably more co-morbidities including CVD than those with Non-Medicaid insurance, which impacts the short-term and long-term effects of malignancy and its treatments on CVD outcomes [19].

It is particularly concerning that this insurance disparity may further contribute to and complicate racial disparities in healthcare outcomes, as non-white patients were disproportionally enrolled into Medicaid or were uninsured due to their socioeconomic status. Furthermore, the insurance disparity in CVD mortality among cancer survivors was actually more prominent as compared to all cause or cancer specific mortality. This highlights the emerging need for greater attention to the cardiac aspects of care in cancer survivors and warrants close collaboration with the cardio-oncology, primary care and oncology physicians to achieve high quality care to narrow the current gap in outcomes.

Another interesting finding of our study was that cardiovascular outcomes of cancer patients with Medicaid were not superior to patients without insurance. Medicaid patients may have lower income levels compared with patients without any insurance. It is also likely that some uninsured patients didn’t buy their health insurance because they were previously healthy before their cancer diagnosis and had less CVD risk factors. These uninsured patients may be younger and fitter than Medicaid patients [20]. However, it is also possible that Medicaid doesn’t provide adequate health coverage to this participially vulnerable populations, especially those with CVD or at high risk of CVD events, because of its lower reimbursement levels and limited access to high-quality, multidisciplinary care as compared to Non-Medicaid insurance. Admittedly, SEER database only collects patients’ insurance information at cancer diagnosis instead of throughout the treatment course and thus we can’t analyze patients whose insurance status may have changed after their cancer diagnosis.

While our study included a large population of cancer patients across the United States, it is subjected to some limitations associated with the data source (SEER database). First, participants over age 65 were excluded from analysis because SEER registries don’t record the detailed insurance information except for Medicare in this population. Information on insurance from cancer registries is widely used for epidemiological and health service research but it is limited by the possibility of changing prior insurance or enrolling insurance after cancer diagnosis. Second, SEER registries use death certificate to determine the cause of death instead of autopsy or electronic chart information, which may introduce misclassification bias. Death from CVD may thus be over/underestimated. Third, SEER registries have no information on patient’s co-morbid diseases, performance status, and smoking behaviors. We can’t identify if CVD risk factors were related to or exacerbated by the patients’ malignancy or its treatments. Pre-existing CVD may interact with cancer and its treatment and thus contribute to higher risks of both long-term and short-term cardiovascular side-effects. These unmeasurable confounders may have influenced our findings.

## Conclusions

Cancer patients with Non-Medicaid insurance have significantly lower CVD mortality than those with no insurance or Medicaid. The insurance disparity remained significant regardless of type of CVD, cancer site, year of diagnosis and follow-up time. While Cardio-oncology develops protocols to follow and aid in the treatment of cancer patients through their cancer treatment and beyond, recognition and awareness of these insurance disparities in CVD mortality among cancer patients is warrantied to alleviate disparities in outcomes across insurance status.

## Supplementary Information

**Additional file 1.**

## Data Availability

All data generated or analyzed during this study are included in this published article.

## Abbreviations

CVD: cardiovascular disease
SEER: Surveillance, Epidemiology and End Results
SMRs: Standardized mortality ratios
HR: Hazard Ratios
MUS: Medicaid or uninsured status
ACA: Affordable Care Act
ICD: International Classification of Diseases
