# Supplementary material for: Insurance disparity in cardiovascular mortality among non-elderly cancer survivors
Source: Cardiooncology. 2021 Mar 20;7:11. doi: 10.1186/s40959-021-00098-8 (PMC7980587; doi:10.1186/s40959-021-00098-8)
Supplement: Supplementary file 1 — Additional file 1. [file 40959_2021_98_MOESM1_ESM.docx]

Supplementary Figure

Potentially eligible patients

N=4453203

Did not meet inclusion criteria N=3654001

Confirmed eligible/Included in the study

N=771281

Analyzed

N=768055

Completing follow-up

N=768055

No information on survival/Cause of Death N=3226

Excluded: Lack of insurance information N=27921

Assessed for Eligibility

N=799202
